# National Trends and Hospitalizations Related to Pancreatic Cancer in Acute Pancreatitis Patients: A Nationwide Inpatient Sample Study

**DOI:** 10.7759/cureus.5155

**Published:** 2019-07-17

**Authors:** Vijay Gayam, Jasdeep S Sidhu, Amrendra Mandal, Pavani Garlapati, Sreedhar Adapa, Venu Madhav Konala, Srikanth Naramala, Eric O Then, Srikanth Maddika, Vinaya Gaduputi

**Affiliations:** 1 Internal Medicine, Interfaith Medical Center, Brooklyn, USA; 2 Nephrology, The Nephrology Group, Visalia, USA; 3 Internal Medicine / Hematology and Oncology, Ashland Bellefonte Cancer Center, Ashland, USA; 4 Rheumatology, Adventist Medical Center, Hanford, USA; 5 Gastroenterology, St. Barnabas Hospital Health System/Albert Einstein College of Medicine, Bronx, USA; 6 Internal Medicine, St. Barnabas Hospital Health System, Bronx, USA

**Keywords:** pancreatic cancer, acute pancreatitis, national inpatient sample, the international statistical classification of diseases (icd-10)

## Abstract

Background

Pancreatic cancer (PC) is one of the common cancers in the United States (U.S.) and is associated with high mortality and morbidity. In spite of the modest improvement in survival, cancer care costs including PC continue to rise and inpatient costs contribute a significant chunk to cancer care, which is often ignored. Acute pancreatitis (AP) is a rare manifestation of PC. This study aims to determine the national trends and associated health care utilization of PC patients hospitalized with AP in the U.S.

Methods

We used National Inpatient Sample (NIS) to extract data for patients hospitalized with a primary diagnosis of PC in AP in 2016 using International Classification of Diseases, 10th revision, and Clinical Modification (ICD-10-CM) codes. The analysis included disease etiologies, age, race, sex, hospital region, hospital size, institution type, mortality, length of hospital stay (LOS), and commonly associated comorbidities were correlated.

Results

There were 250 patients with a discharge diagnosis of PC in patients admitted with AP. Most of the patients were whites (76.6%) with the mean age of 39.42 ± 2.51 years, had Medicare (63.26%) as primary insurance, were from Southern region (46%) and had higher Charlson comorbidity index (CCI) (76.00% with CCI > = 3). The mean hospital charges were $48,462.13, and mean LOS was 5.24 days. The LOS was significantly impacted by race, hospital region, endoscopic retrograde cholangiopancreatography (ERCP), and comorbidities such as dementia, smoking, and seizure. Out of the 250 patients admitted with PC, 245 patients (98%) were discharged alive.

Conclusions

Our study shows a downward trend in LOS, hospital charges, and in-hospital mortality as compared to other studies despite PC and AP presenting together versus PC with or without other etiologies.

## Introduction

Pancreatic cancer (PC) is the twelfth most common cause of cancer in the United States (U.S.) and is the fourth most common cause of cancer-related deaths [1-3]. PC is associated with high morbidity and mortality and with an aging population as well as changing demographics; the incidence of PC is estimated to rise over time and is projected to be the second most common cause of cancer-related deaths by 2030 [4]. Recently, it has been described that acute pancreatitis (AP) is an early symptom of PC. Mujica et al. reported that the one-year overall survival rate was 28% in patients with PC presenting with AP and 20% in patients with PC without AP [5].

Approximately 53,000 U.S. populations were anticipated to be diagnosed with PC, and 42,000 were expected to die from this disease in 2016 [3]. The five-year survival rate from PC between 2005 and 2011 was about 8%, and survival has improved only slightly in the last 35 years [3]. About 20% of the patients who undergo surgery would survive from cancer. However, only 15%-20% of PC patients are candidates for surgery [6-7].

Surgical candidates are likely to undergo adjuvant chemotherapy after surgery, and patients with unresectable tumors may be candidates for chemotherapy or chemoradiation therapy; all of these contribute significantly to morbidity [8-9]. The curative-intent pancreaticoduodenectomy, or Whipple procedure, is the most common surgery performed. However, this surgery is associated with high morbidity, extended the in-hospital stay, and has a mortality rate of 1%-5% [10]. PC patients have frequent hospitalizations for the establishment of diagnosis, recovery from surgery, and complications related to cancer, surgery, or chemotherapy [11].

The most common risk factors associated with AP are gallstones, alcohol, and smoking [12-15]. Another major contributor to increasing detection and diagnosis of AP is more frequent laboratory testing [16]. The link between AP and PC have been insufficiently investigated. To further investigate this issue, we extracted the data from the Nationwide Inpatient Sample (NIS), the largest inpatient database in the U.S. and analyzed the overall trends such as mortality, length of hospital stay (LOS), commonly associated comorbidities regarding PC in AP.

## Materials and methods

Data source

Data was collected using the NIS database for hospitalized patients with PC and AP for International Classification of Diseases, 10th revision, and Clinical Modification (ICD-10-CM). Related etiologies and associated diagnoses were queried from secondary diagnoses using respective ICD-10-CM codes.

Patients and outcomes

Patient-level variables included age, sex, race, median household income for patient’s zip code (quartiles), and insurance status. Race/ethnicity was categorized as white, black, Hispanic, and others. Insurance status was categorized as Medicare, Medicaid, private insurance, and uninsured/other based on the primary payer listed on the discharge record. Hospital location and teaching status were combined into a single variable with the following categories: rural, urban non-teaching, and urban-teaching hospital. The U.S. Census Bureau classified hospital regions as Northeast, Midwest, South, or West. The presence of a primary diagnosis of PC and AP-related etiologies, disease conditions, and clinical outcomes were evaluated in 2016.

The primary outcome of interest was the frequency of PC-related LOS, and total hospitalization costs and charges.

Statistical analysis

Categorical variables were tested for statistical significance with Chi-square analysis. Continuous variables were tested using the t-test. The mean and standard error were calculated for all continuous outcomes, and frequency counts and percentages for categorical outcomes. Statistical significance was defined by p <0.05.

## Results

**Figure 1:**
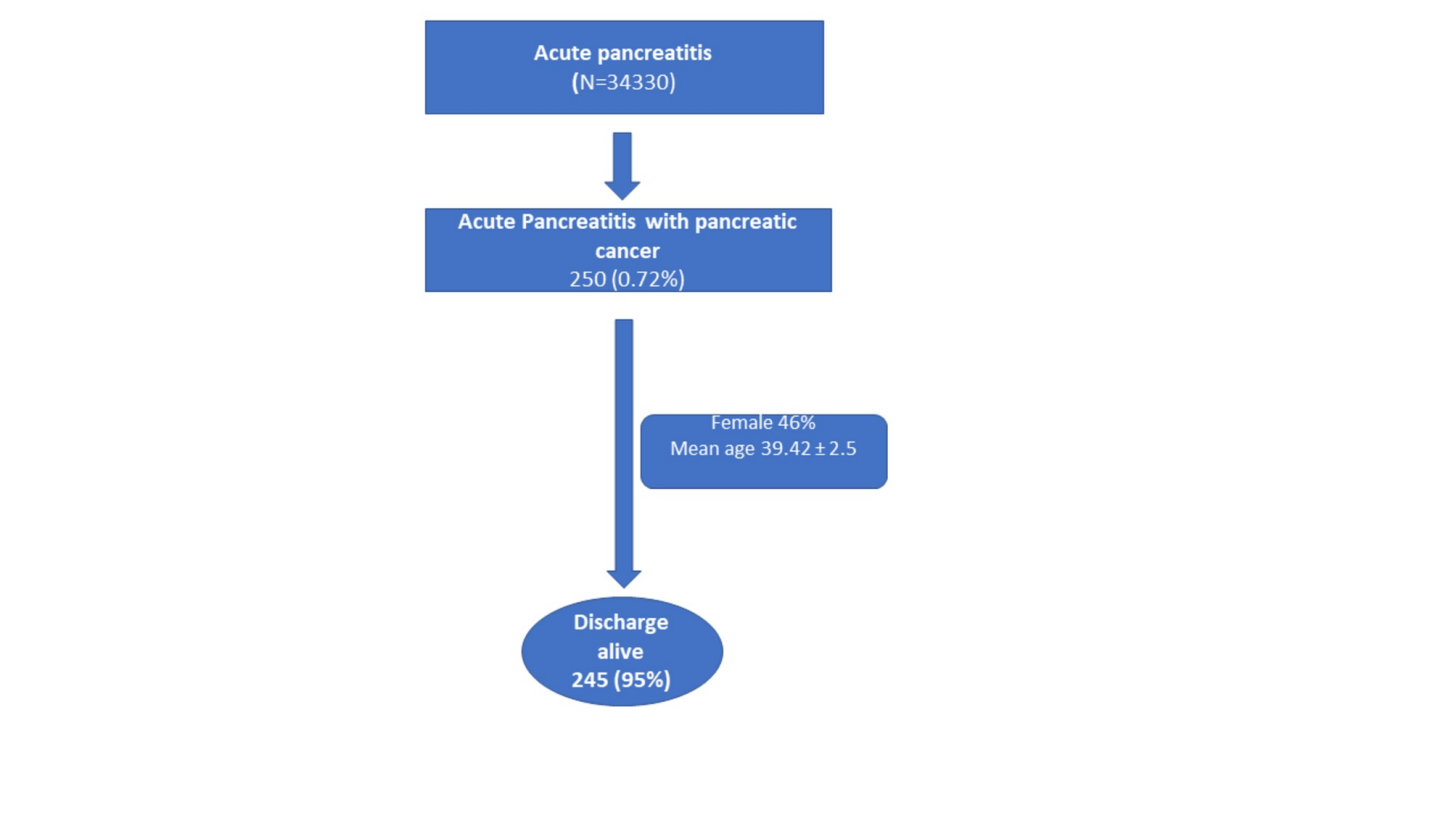
The flow chart of the study population with acute pancreatitis and pancreatic cancer

The flow chart of the study population with AP and PC is shown in Figure 1.

We reviewed the National Inpatient Sample (NIS) for 2016 in patients with PC presenting with AP. There were 250 patients with a diagnosis of pancreatic cancer among 34330 patients admitted with AP.

The clinical characteristics of the patients included in the study are shown in Table 1. The mean age of the population was 39.42 ± 2.51 years. The majority of patients were white (76.60%), female (46%), had Medicare (63.26%) as their main insurance, live in the Southern region of the U.S. (46%) and had higher Charlson comorbidity index (CCI) (76% with CCI > = 3).

**Table 1:** Characteristics, etiologies, and associated conditions of acute pancreatitis and pancreatic cancer in 2016

Variable	Acute Pancreatitis with pancreatic cancer	Acute pancreatitis (Total)
Total sample size	N= 250	N=34330
Female %	46.00	49.53
Mean age in years	39.42 ± 2.51	41.52 ± 1.30
RACE		
Whites %	76.60	65.11
Black %	6.38	16.97
Hispanic %	10.64	12.36
Asian/Pacific Islander %	4.25	1.99
Native American%	2.13	0.71
Other	0	2.84
Weekend Admissions %	26.00	26.20
Weekday admissions %	74.00	73.80
Insurance provider (%)		
Medicare %	63.26	36.94
Medicaid %	10.20	22.58
Private %	24.50	33.11
No insurance %	2.04	7.37
Charlson comorbidity index %		
0 %	0	38.18
1 %	0	28.27
2 %	24.00	15.40
3 or more %	76.00	8.15
Patient residence%		
Hospital Region %		
Northeast	14.00	14.96
Midwest	26.00	23.04
South	46.00	44.25
West	14.00	17.74
Teaching hospital %	70.00	44.11
Non-Teaching Hospital %	30.00	55.89
Urban Hospital location %	96.00	85.54
Non-Urban Hospital Location	4.00	14.46
Co-morbidities (%)		
Diabetes	44.00	33.20
Peripheral Vascular Disease	6.00	4.44
Chronic obstructive pulmonary disease	14.00	17.00
Renal Disease	6.00	11.76
Liver Disease	12.00	14.31
Cerebrovascular Disease	4.00	1.50
Coronary Artery Disease	8.00	4.41
Dementia	4.00	2.21
Peptic Ulcer Disease	0	1.83
Congestive Heart Failure	12.00	6.80
Rheumatoid Disease	6.00	2.30
HIV Disease	4.00	0.6
Smoking	4.00	6.40
Alcoholism	2.00	3.00
Gallstones	4.00	3.07
hypertriglyceridemia	4.00	7.79
Hypercalcemia	4.00	0.91
Cystic Fibrosis	0	0.1
Hypertension	72.00	45.56
Dyslipidemia	48.00	28.90
Seizure	2.00	3.50
Cardiac Arrhythmia	12.00	4.08
Psychiatric Disorder	0	1.49

There were few factors significantly associated with LOS such as the intervention with endoscopic retrograde cholangiopancreatography (ERCP) to relieve the obstructions as a possible cause for AP and the comorbidities such as dementia, smoking, and seizures also impacted the LOS as shown in Table 2.

**Table 2:** Factors affecting length of hospital stay in patients with acute pancreatitis presenting with pancreatic cancer

Variable	Coefficient	95% confidence interval	p-value
RACE			
Hispanic	7.17	3.25 – 11.08	0.00
Asian/Pacific Islander	7.49	1.53 – 13.45	0.01
Native American	5.08	3.71 – 6.45	0.00.1
HOSPITAL REGION			
Midwest	4.77	0.45 – 9.10	0.03
South	2.46	(-0.34) – 5.26	0.09
West	5.89	2.19 – 9.59	0.00
Endoscopic retrograde cholangiopancreatography (ERCP)	5.00	1.14 – 8.84	0.01
Co-morbidities (%)			
Dementia	-10.21	(-13.60) – (-6.81)	0.00
Smoking	2.93	1.61 – 4.23	0.00
Seizure	9.70	6.39 – 13.00	0.00

The mean LOS was 5.24 days, the mean hospital charges were $48,462.13, and hospital cost was $10,932, as shown in Table 3.

**Table 3:** Resource utilization

	Acute pancreatitis with pancreatic cancer	Acute pancreatitis
Total Admission	N= 250	N= 34324
Discharged Alive (%)	245 (98%)	34129 (99%)
Mean LOS (Days)	5.24	3.79
Patients undergoing ERCP	30 (12%)	1170 (3.40%)
Mean Total Charge ($)	48462.13	30725.16
Mean Total Cost ($)	10932	7684.92
Total LOS (Days)	1310	130087

## Discussion

There are insufficient published data available regarding the prevalence and clinical characteristics of patients with PC presenting with AP, unlike chronic pancreatitis. To our knowledge, this is the largest sample of PC in patients with AP using ICD-10 for the year 2016. There has been varied reported incidence of AP in PC patients in the range between 6.8% and 13.8% [17].

The underlying mechanisms and the natural course of this disease are unclear. There is a possible explanation; including obstruction of pancreatic ducts, which resulted in the dilatation of the main pancreatic duct and activated pancreatic enzymes [5,18]. Likewise, Kimura et al. reported that PC might produce some chemical mediators, which may be responsible for AP [17]. AP can, however, occur in the absence of main pancreatic duct obstruction as explained by Pelletier et al. who reported that the slow growth of PC might not narrow the main pancreatic duct; thus, AP may not frequently occur [19].

In the study by Munigula et al., AP is a rare manifestation of PC, and therefore, these groups of patients are usually misdiagnosed. Generally, patients with PC is diagnosed in the first year after AP [20]. Minato et al. hypothesized that pancreatic inflammation might mask the evidence of an underlying lesion in the pancreas or a small-sized tumor may hinder an early diagnosis of cancer [21].

Kirkegard et al. in a Nationwide Matched cohort study found that patients admitted with AP have a twofold increased risk of PC compared with the general population, even after ten years of follow-up [22].

The National Cancer Institute published the estimated incidence for 2016 as 14.1/100,000 (a rise from 12.1/100,000 in 2005) [23]. Growing estimations are probably due to a surge in the number of patients (>65 years of age) and an increased incidence of PC likely among the immigrant populations, which are not only growing in number but may also have a higher rate of PC.

Bhandari et al. in a NIS from 2007 to 2011 observed that LOS decreased by 0.6 days per admission, the mean hospital charge increased by 24%, stating there have been improvements in recovery however at the cost of financial burdens [24]. The rise in hospital charges despite the decline in LOS could be attributed to increases in the use of treatment and health care resources. This might have translated into the rise in the survival of the patients. Similarly, Wang et al. demonstrated that LOS decreased from 10.7 days in 2000 to 9.1 days in 2010 [11].

The present study shows that there is a lower LOS of 5.24 days in 2016. However, the studies of both Bhandari et al. and Wang et al. were on patients with PC while our study is on patients with PC presenting with AP. Present and previous studies, therefore, support the decline in LOS due to PC in the U.S. during the last decades regardless of association with AP.

In our study, mean total hospital charges due to PC in patients with AP were calculated to be $48,462.13. With projected increases in the number of patients with PC presenting with AP from previous studies, the economic burden of PC in the future is significant [11,22-24].

Our study has several limitations; it is retrospective, and some patient might have recurrent admissions and decrease in-hospital mortality may not precisely reflect an actual decline in death as many patients possibly going to hospice or getting treatment as outpatients could not be expressed in the NIS database with the use of ICD-10-CM.

## Conclusions

Our study showed downward trends in LOS, hospital charges, and in-hospital mortality as compared to previous studies despite PC and AP presenting together versus PC with or without other etiologies. AP might be the presenting feature of PC and patients presenting with AP may be considered as a possible diagnostic indicator of PC.
